# Genome-wide association analysis for heat tolerance at flowering detected a large set of genes involved in adaptation to thermal and other stresses

**DOI:** 10.1371/journal.pone.0171254

**Published:** 2017-02-02

**Authors:** Tanguy Lafarge, Crisanta Bueno, Julien Frouin, Laval Jacquin, Brigitte Courtois, Nourollah Ahmadi

**Affiliations:** 1 CIRAD, UMR AGAP, Montpellier, France; 2 International Rice Research Institute, Los-Banos, Philippines; Institute of Genetics and Developmental Biology Chinese Academy of Sciences, CHINA

## Abstract

Fertilization sensitivity to heat in rice is a major issue within climate change scenarios in the tropics. A panel of 167 *indica* landraces and improved varieties was phenotyped for spikelet sterility (SPKST) under 38°C during anthesis and for several secondary traits potentially affecting panicle micro-climate and thus the fertilization process. The panel was genotyped with an average density of one marker per 29 kb using genotyping by sequencing. Genome-wide association analyses (GWAS) were conducted using three methods based on single marker regression, haplotype regression and simultaneous fitting of all markers, respectively. Fourteen loci significantly associated with SPKST under at least two GWAS methods were detected. A large number of associations was also detected for the secondary traits. Analysis of co-localization of SPKST associated loci with QTLs detected in progenies of bi-parental crosses reported in the literature allowed to narrow -down the position of eight of those QTLs, including the most documented one, qHTSF4.1. Gene families underlying loci associated with SPKST corresponded to functions ranging from sensing abiotic stresses and regulating plant response, such as wall-associated kinases and heat shock proteins, to cell division and gametophyte development. Analysis of diversity at the vicinity of loci associated with SPKST within the rice three thousand genomes, revealed widespread distribution of the favourable alleles across *O*. *sativa* genetic groups. However, few accessions assembled the favourable alleles at all loci. Effective donors included the heat tolerant variety N22 and some Indian and Taiwanese varieties. These results provide a basis for breeding for heat tolerance during anthesis and for functional validation of major loci governing this trait.

## Introduction

Spikelet sterility in the rice crop is becoming a burning issue in the context of global warming since it may occur as soon as air temperature reaches 33°C at time of anthesis [1] even if it is for less than an hour of exposure [2], a situation which can already be encountered in many rice growing areas [3]. Meanwhile, the linear tendency of global warming was 0.74°C for the period 1906–2005 [4]. Because of higher recurrence of extreme high-temperature events and a projected global average surface temperature increase of 1.5 to 4.8°C by 2100 [5, 6], yield decrease in the 2^nd^ half of the century is predicted to be even stronger in the tropics than in the temperate areas [7]. Considering also a simulated increase in annual mean of maximum temperature during the period 1990–2050 of 0.5 to 1.0°C in the northern and central part of South-East Asia, and of 1.0 to 1.5°C in the southern part [8], spikelet sterility due to heat in rice will become more dramatic worldwide in the near future.

Grain yield can be strongly reduced by spikelet sterility under heat [9, 10], with damages being highly dependent on air relative humidity [11]. Yield losses of about 5 million t of paddy rice were recorded along the Yangtze River in China in 2003 [12, 13] due to extreme high day temperature episodes. Yield losses of 25% were measured in some areas of the Kanto and Tokai regions in Japan in 2007 after unusual temperatures exceeding 35°C around heading stage [14]. Yield losses will be even worst in the near future, considering not only heat but also more frequent stomatal closure, as a consequence of higher CO_2_ concentration level and more frequent water deficit, acting indirectly on plant temperature [15, 16]. Indeed, Krishnan et al [9] have simulated yield reduction in Eastern India with a temperature increase of 1 to 5°C, despite the increase in atmospheric CO_2_ concentration with the higher temperatures.

Some adaptation strategies to heat in rice have been recently described [17, 18]. As an escape strategy, plants already have the ability to adjust the time of the day of anthesis depending on their sensitivity to climate conditions during the 7-days period preceding anthesis [17]: anthesis is advanced earlier in the morning if conditions during the seven preceding days are hot and humid, allowing the plants to escape the hottest time of the day. As an avoidance strategy, in dry air environments, the transpirational cooling ability of the plant can lower panicle temperature by up to 10°C compared to air temperature and so stabilize spikelet fertility by maintaining panicle temperature below the critical threshold [18, 19]. This is facilitated when top leaves are long and erect and protect the panicle from direct sunshine [18]. This provides an avenue to increase fertility under heat although this cooling ability is strongly limited under high humidity conditions [11, 18]. Some genetic variability was reported in the cooling capacity of the panicle through its transpiration and that of the surrounding leaves [20]. Spikelet sterility is confirmed to be well correlated to panicle temperature [18, 21], and increased by 7% per additional degree Celsius in IR64, and by 2.4% in Azucena varieties, when panicle temperature was above 29.6°C and 33°C, respectively [2].

Sterility is, however, still a major concern in rice cultivation areas despite the occurrence of escape and avoidance strategies because most of the concerned regions are characterized by humid conditions, limiting the incidence of transpirational cooling, then panicle temperature reduction [11, 18]. In addition, with erratic rainfall patterns, increasing pressure on irrigation water, and increasing occurrence of combinations of abiotic stresses such as heat, drought and salinity in farmers’ fields [22], transpiration cooling shall become even lower because of partial stomatal closure under future climates, hence, drastically increasing the vulnerability of rice in the most productive regions. To cope with future climate scenarios, developing rice varieties with tolerance to heat under varying amounts of humidity is vitally important.

High temperature during rice anthesis results in a reduced number of anthesing (opened) spikelets, poor anther dehiscence, poor pollen viability (reduced number of germinated pollen grains on the stigma) and reduced length of pollen tubes [19, 23, 24]. These morpho-physiologic events are associated with quantitative and qualitative biochemical changes: while moisture content and total protein concentration of pollen grains decrease as well as the membrane stability indices, sugar content increases. Proteins with the highest molecular subunits (more than 66 kDa) are disintegrated into low molecular mass polypeptides (below 43 kDa), leading to the loss of pollen viability [25].

Large genetic variation exists in the spikelet sensitivity to high temperature damage. Accessions tolerant to high temperature have been identified in all major rice genetic groups [15, 26, 27, 28, 29]. Among them, N22, an Indian landrace belonging to the *aus* genetic group, is one of the most heat-tolerant varieties. For instance when submitted to 38°C for 6 hours during anthesis, N22 maintained a spikelet fertility of 71% while the fertility was of 48% for the moderately tolerant *indica* variety IR64 and of only 18% for the tropical *japonica* variety Moroberekan [29]. High heat tolerance during anthesis was also reported for Giza178, a *japonica* variety from Egypt [30].

During the last decade several studies aiming at dissecting the genetic bases of spikelet sensitivity to high temperature have been reported. Using a bulk-segregant analysis approach in a F_2_ population of 279 individuals derived from cross between the heat-tolerant cultivar 996 and the sensitive cultivar 4628, Zhang et al [31] identified two SSR markers (RM3735 on chromosome 4 and RM3586 on chromosome 3) significantly associated with heat tolerance, accounting for 17% and 3% of the total variation, respectively. Later on, using linkage mapping approach in recombinant inbred lines derived from the same cross, Xiao et al [32] detected two QTLs affecting spikelet fertility under high temperature on chromosome 4 (RM5687—RM471) and chromosome 6 (RM190—RM225). They explained 15.1% and 9.3% of the total phenotypic variation, respectively. Jagadish et al [23] identified eight QTLs associated with spikelet fertility under heat on six different chromosomes within a population of 181 recombinant inbred lines derived from a tolerant (Bala) and a susceptible (Azucena) parent. The most significant heat-responsive QTL mapped on chromosome 1 (38.35 Mb) and explained 18.1% of the phenotypic variation. Ye et al [33], using BC1F1 and BC2F2 populations derived from a cross between N22 and IR64 varieties, detected two putative QTL, located on chromosome 1 (qHTSF1.1) and chromosome 4 (qHTSF4.1), explaining 12.6% and 17.6% of the total variation, respectively. More recently, Ye et al [30] mapped four QTLs (qHTSF1.2, qHTSF2.1, qHTSF3.1 and qHTSF4.1) in a F2 population of IR64/Giza178, and two other QTLs (qHTSF6.1 and qHTSF11.2) in a F2 population of Milyang23/Giza178. The QTL on chromosome 4 (qHTSF4.1) colocalised with the QTL on chromosome 4 previously identified in the IR64/N22 population. However, the confidence intervals of those QTLs, at best between 0.8 and 1.8 Mb [30], are still too large to search for candidate genes.

At the other end of the spectrum of genetic analyses, using proteomic approach Jagadish et al [34] reported much higher accumulation of small heat shock proteins (sHSP) in N22 variety compared with the heat-sensitive variety Moroberekan, and the moderately heat-tolerant variety IR64, when submitted to high temperature during anthesis. Likewise, semi-quantitative RT-PCR based gene expression analysis, targeting heat shock proteins (Hsps) and heat shock transcription factors (Hsfs), identified N22 as the variety with the highest level of expression for most of the Hsps and Hsfs genes tested [35]. Several other studies have analyzed differential gene expression in rice subjected to high temperature [36, 37, 38] but they did not focus on the reproductive stage and the resulting spikelet sterility.

Genome-wide association studies (GWAS) has emerged as a tool to resolve complex trait variation down to the sequence level by exploiting historical and evolutionary recombination events at the population level [39, 40]. As an alternative to linkage analysis, association mapping offers three advantages, (i) increased mapping resolution, (ii) reduced research time, and (iii) greater allele number [41]. The approach was already successfully applied in rice to dissect the genetic bases of complex traits such as plant development and agronomic performance [42], root development [43], rice grain concentration in metalloids and micronutrients [44], drought tolerance [45], grain shape and other grain related traits [46]. The simplest form of GWAS is the marker-by-marker analysis, but methodology for more complex models have recently been developed [47].

To date, GWAS approach has not been used for the exploration of loci controlling tolerance to high temperature in rice. Here, we report application of three GWAS methods to detect QTLs for spikelet fertility under high temperatures during the anthesis process, and for several other phenotypic traits, in a diversity panel of 167 *indica* accessions genotyped with 13,162 SNPs. To minimize any bias when measuring spikelet fertility, only the top half of the panicles that had effectively experienced heat at time of anthesis were sampled and analyzed. Co-localizations with previously identified QTLs and with candidate genes were analyzed with the aim of connecting those scales of analysis and providing a unified picture of the genetic bases of spikelet tolerance to high temperature.

## Materials and methods

### Plant material

The plant material was composed of 167 accessions extracted from a diversity panel of 201 traditional and improved *indica* accessions plus six *aus* accessions including the heat tolerant variety N22 (S1 Table). These accessions were chosen so as to cover the largest geographical origins and products of the major international lowland rice breeding programs. Seeds of the material were obtained either from Centre de Ressources Biologiques Tropicales de Montpellier (http://golo.cirad.fr/FR/) or from AfricaRice, CIAT or IRRI gene banks. The accessions were seed increased during two generations in Cirad Montpellier greenhouse, using single seed descent to make sure they were homogeneous.

### Phenotyping

#### Experimental setup

The experiment was conducted in a greenhouse and in controlled growth facilities of the International Rice Research Institute (IRRI), Los Baños, Laguna, Philippines (14°11′N, 121°15′E), from July to November of 2009. Seeds of the 167 accessions were germinated in Petri dishes in a germination chamber at 32°C. After 2 days, when the coleoptiles reached 2–3 mm length, five pre-germinated seeds of each line were sown in a 6.5 l PVC pot containing 7.0 kg soil with 11.1 g organic C kg^-1^, and 1.4 g total N kg^-1^, with a pH of 6.5 and particle size distribution as 33% clay, 38% silt, and 29% sand. Fertilizers were applied in each pot through 0.16 g P as solophos, 0.16 g KCl and 0.06 g ZnSO_4_ at sowing, and 0.16 g N as urea at 10, 41 and 56 days after sowing. Thinning was done to two seedlings per pot one week after sowing and to one seedling two weeks after sowing. Pots were kept flooded from right after thinning until maturity.

Pots were arranged within three separated blocks, each block containing two replicates of each of the 167 lines. The first block, B1, corresponded to the T1 treatment (control), the second one, B2, to the T2 treatment (heat at flowering), and the third one, B3, to an extra set for morphological measurements. All three blocks were grown in a greenhouse without specific climate control until few days before panicle initiation (35 days after sowing) with a constant distance between plants of 30 cm (density of 44 pl m^-2^). At that time, the three blocks were moved to one highly controlled greenhouse compartment each (soil dimensions of 9.7 m by 4.4 m) equipped with a continuous ventilation facility, and grown under 22/28°C night/day thermal conditions. The plants of the three blocks were maintained under these conditions until maturity, except plants of B2 that were subjected to 38°C during 6 days at time of flowering, and plants of B3 that were used for destructive and non-destructive measurements at flowering. In the case of B2, the plants were grown from 8 am to 2 pm (time range that includes the whole period of the day when anthesis occurs) during 6 consecutive days, in an outdoor controlled growth facility (soil dimensions of 1.3 m by 1.3 m) equipped with a continuous ventilation system that maintained a temperature of 38°C in the vicinity of the rice panicles. This treatment started one day after anthesis was first observed on the main tiller.

#### Plant measurements

Accession-dependent traits (plant and panicle architectural patterns, heading date) were measured in the B3 block. Heading date was reported as the date when the panicle of the main tiller appears on top of the stem (DTHD). At flowering, defined as the time when anthesis of 50% of the panicle has occurred, plant height (PTHT) was measured from the soil surface to the tip of the highest leaf when stretched; the number of live tillers (TINB) and tillers bearing a panicle (PNNB) were counted. Plant samples were separated into three entities, green leaf blades, dead leaf blades, and stems plus panicles. One leaf was considered as green if more than half of its surface was still green. The total leaf area (LFAR) of the green blades was measured with a leaf area meter (LI-3100C Area Meter, Li-Cor, Lincoln, NE, USA). Dry matter of each entity, green leaf blades (GLFDW), dead leaf blades (DLFDW), and stems plus panicles (ST+PNDW), was determined by weighing the material after drying during 72 h at 70°C. Shoot dry weight (SHDW) was calculated as the sum of GLFDW, DLFDW, and ST+PNDW. Specific leaf area (SLA) was calculated as the leaf area divided by the corresponding leaf dry weight. Panicle maximal width (PNDM) and position compared with the tip of the highest leaf (PNPOS) was measured for the panicle of each main tiller. Angles of the flag leaf of the main tiller (FLFAG) and the leaf immediately below it (LFAG) were measured with reference to the vertical. Filled and unfilled grains were separated at a flow rate of 4m^3^/s using a Seedburo blower (KL-1205, Seedburo, Chicago, IL, USA) and counted. Spikelet sterility under controlled condition (SPKSTc) was computed as the ratio of the number of unfilled spikelets above the total number of spikelets.

Treatment-dependent traits were measured from each of the other two blocks. The number of green leaves of the main tiller of each entry in each treatment was counted at flowering, and at 16, and 21 days after flowering. The rate of leaf senescence 16 and 21 days after flowering was then calculated as the ratio of the reduction in green leaf number between flowering and 16 and 21 days later, respectively, above the green leaf number at flowering (LFSNS 16, LFSNS 21). Panicle length (PNLT) was measured at maturity from the neck of the panicle, and the rate of panicle exertion (PNEX) at flowering was estimated as the ratio of the visible panicle length at flowering above panicle length at maturity. Flowering duration (HDLT) was calculated as the difference between the day when the last tiller of the plant finished flowering and the day when the first tiller of the plant started to flower. To address spikelet sterility measurement, each single panicle subjected to T2 treatment, for which all the spikelets were exposed to heat at anthesis (considering the six consecutive days of exposure), was tagged with respect to the day when anthesis started. All corresponding panicles that emerged on the same day in the T1 treatment were also tagged. At harvest, only the tagged panicles from both T1 and T2 treatments (that varied from 4 to 9 panicles depending on the genotype) were sampled and cut into two equal pieces (top and bottom parts). Filled and unfilled grains considering the top half of the panicles only (to avoid including unfilled grains because of lack of carbohydrate supply) were separated at a flow rate of 4m^3^/s and counted. Spikelet sterility under heat stress (SPKST) was computed as the ratio of the number of unfilled spikelets above the total number of spikelets of the top half of the panicles that fully flowered during the 6-day period within T2 treatment. The 2 ArcSin Square root transformation was applied to SPKST data expressed as percentage, for association analysis.

### Genotyping

Genotyping was conducted at Diversity Arrays Technology Pty Ltd. (Australia) using a method of genotyping by sequencing (GBS) previously described by Courtois et al [43]. Briefly, a combination of *PstI*/*TaqI* restriction enzymes was used to reduce the genome complexity. The sequences were trimmed at 69 bp (5 bp of the restriction fragment plus 64 bases, with a minimum quality score of 10). An analytical pipeline developed by DArT P/L was used to produce DArT tables (corresponding to the presence/absence of any given sequence) and SNP tables (corresponding to single nucleotide polymorphisms within the 69 bp sequences). The position of each marker on the rice genome was determined by aligning the sequences to the Os-Nipponbare-Reference-IRGSP-1.0 pseudomolecule assembly [48]. Sequences that did not align on the Nipponbare sequence or aligned in several positions were discarded from the initial dataset, as well as markers with more than 20% missing data. For each marker the corresponding RAP-DB annotation (http://rapdb.dna.affrc.go.jp) was retrieved and, when it corresponded to a gene, the position regarding gene attributes (intron, exon, 3' or 5' UTR) was determined.

To constitute the matrix that was used for GWAS, markers with frequency of minor allele (MAF) below 2.5% were discarded, and the few heterozygous loci were replaced by missing data. Then missing data were imputed using Beagle v3.3.2 [49]. Finally, when the distance between two consecutive markers was below 1 kb, the marker with the lowest MAF was discarded.

The phenotypic and genotypic data are available at http://tropgenedb.cirad.fr/tropgene/JSP/interface.jsp?module=RICE, (Choose Tab Studies) as “ORYTAGE_indica_Heat-tolerance_167-accessions_21-traits”, and “ORYTAGE_indica_GBS-genotype_167-accessions_13160-SNP”

### Analysis of phenotypic data

The standard score of phenotypic data for 20 traits and 167 accessions was submitted to principal component analysis (PCA) and to factorial discriminant analysis (FDA) using XLSTAT package. In the later analysis, the membership in subpopulations identified by Structure analysis on genotypic data (see below) was used as categorical variables.

### Analysis of population structure

To analyze population structure, a sub-set of 825 SNP markers with less than 3.5% missing data, MAF>5%, and distant from one another of at least 100 kb was selected from the initial matrix. The population structure of the panel of 201 accessions was analyzed using the model-based approach of Structure v3.2. [50] which was run with the following parameters: haploid data, possibility of admixture, correlated frequencies for a number of subpopulations (K) varying from 1 to 10 and 10 runs per K value. To determine the number of subpopulations, both Evano's and DAPC methods were used as described in [43]. Once the likely number of subpopulations determined, each accession was assigned to one of those subpopulations, if the proportion of its inferred ancestry derived from a subpopulation was above 2/3 or 67%. Otherwise the accession was considered as admixed. A distance-based clustering method was also implemented: an unweighted NJ tree based on a simple matching matrix was constructed using DarWin v6 [51]. The subpopulation assignments driven from structure analysis were projected on this tree.

### Linkage disequilibrium

The speed of decay of linkage disequilibrium (LD) in the panel was estimated by computing r^2^ between pairs of markers on a chromosome basis using Tassel 5.2 software [52], and then averaging the results by classes of distance using XLSTAT.

### Association mapping

First, a single marker regression based association analysis (Sm-GWAS) was performed for all phenotypic traits under a Mixed Linear Model (MLM) where markers and population structure (Q matrix) effects were considered as fixed and the kinship effect (K matrix) was considered as random. The MLM was run under the exact method option of Tassel 5.2 software [53], where the additive genetic and residual variance components–the random factors of the mixed model–are re-estimated for each SNP. For each SNP tested, Tassel 5.2 computed a p-value, the log likelihoods of the null and alternative models, and the fixed-effect weight of the SNP with its standard error. The threshold to declare significant an association was set at a probability level of 1e-05.

Second, in order to ascertain the results of the single regression based association analysis eight phenotypic traits chosen for the importance of their discrimination power within the panel, were submitted to association analysis using two other methods, namely haplotype based GWAS and simultaneous fitting of all markers.

Haplotype based GWAS (Hap-GWAS) consists in testing the effect of haplotypes in sliding windows of SNPs (instead of individual SNP) across the genome for their association with phenotype. It is based on hypothesis that marker haplotypes are in greater LD with the QTL alleles than single markers [54]. If this is true, then the r^2^ between the QTL and the haplotypes will increase, thereby increasing the power of the experiment [47]. The mixed model we used to test for the presence of a QTL at a given position was Y = X_β_ + Z_h_*h* + Z_u_*u* + Ɛ where β is a fixed effect, which is the overall mean, and X = 1_n_ is a vector of ones. Vector *u* represents the random polygenic effects due to relationships among individuals. Vector *h* represents the random effects of haplotypes and is a function of the number of observed haplotypes at each tested position, which is defined by the center of the sliding window. Haplotype effects are treated as random as it is likely that many haplotypes will have low frequencies due to the small sample size (i.e. 167). The identity by state, between the marker alleles of haplotypes, was used as the allele identity predictor at the QTL. The size of the windows was equal to 6 consecutive markers, whatever the distance between those markers. The restricted maximum likelihood ratio test (RLRT) was used to decide for the existence of association for each tested position. Hap-GWAS was implemented using a script developed by Jacquin et al [54].

GWAS based on simultaneous fitting of all markers involves fitting the models that have been proposed for genomic prediction [55]. The model we chose was the Bayesian lasso [56], which assumes that SNP effects are random and are derived from a double exponential or Laplace distribution. This distribution induces a type of shrinkage of estimates that is size-of-effect dependent, i.e. there is a possibility for SNPs to have a null, a moderate or a large effect. The Bayesian lasso model based GWAS (BL-GWAS) was implemented using BGLR statistical package [57]. BGLR output included the individual marker effects, the estimated posterior mean and standard deviation of the marker effects, the estimated posterior mean of genomic values of N accessions and statistics related to deviance information criterion DIC. To decide for the level of marker effects to be considered as significantly different from zero, we performed a permutation test. For each trait, 1000 permutations were performed and the significance threshold was set as to correspond to less than 0.005%. Moreover, to further ascertain loci with high effect, a robustness test was performed for PTHT and SPKST traits as follows: first, 100 samples were constructed by randomly drawing 75% of the accessions of the panel; then, samples were reanalyzed using the BL-GWAS that was used for the raw data to construct the distribution of the test statistics, i.e. mean and standard deviation of the effects of the 13,160 SNP markers over the 100 simulations. The threshold to declare significant an association was set at the false positive probability level of P< 0.05.

### Analysis of co-localization of significant SNP with known QTLs

To analyze co-localization of significant SNPs detected by GWAS with QTLs reported in literature using progenies of biparental crosses, we used the QTL database developed by Courtois et al [58] and the Gramene QTL database (http://www.gramene.org/). In the case of SPKST, the database used was the one proposed by Ye et al [30], specifically focusing on SPKST resulting from high temperature during anthesis, plus the QTLs those authors have reported in their own paper. To shift from genetic maps to physical maps, the positions of the QTLs were determined by the physical position of the most significant markers linked to the QTLs. To determine the physical positions of the markers, Gramene data or BLAST results based on Gramene sequence information were used.

## Results

### Marker distribution and linkage disequilibrium

The genotypic data set resulting from data cleaning pipeline and imputation, was composed of 13,160 markers (6493 DArT and 6667 SNP markers), which corresponded to an average density of one marker per 29 kb. There were 63 gaps of more than 250kb and nine gaps of more than 500 kb on chromosomes 2, 4, 6, 7, 8 and 11, among which two gaps of approximately 1 Mb (S2 Table). The median MAF varied from 12% (chr 7) to 20% (chr 11) and the overall median MAF was of 16% (S2 Table).

The r^2^ estimate of LD in each of the 12 chromosomes averaged 0.51 for marker pairs whose distance was below 25 kb. The LD reached half its initial value at distance of 125–150 kb between markers and went below 0.1 at distances above 600 kb (S3 Table). There was very little variation between chromosomes for LD features. Given these LD features, the average density of one marker per 29 kb seemed amply sufficient for whole-genome association mapping.

### Panel structure

**Fig 1:**
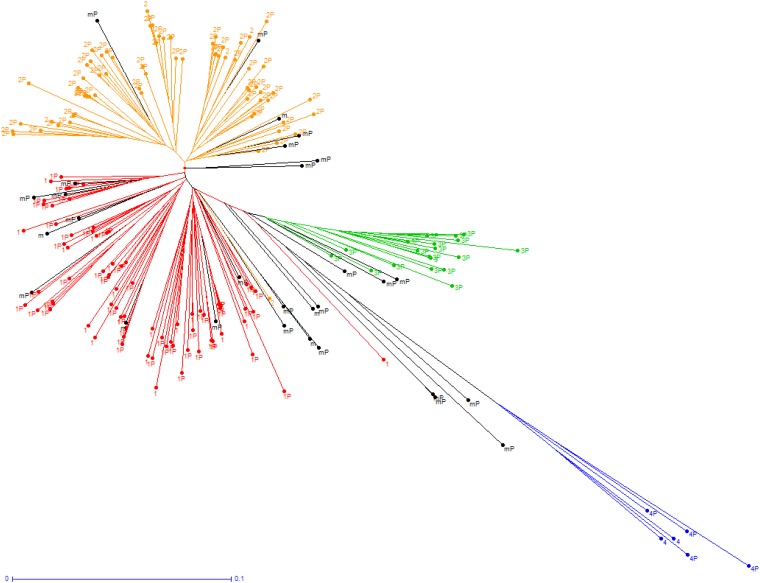
Unweighted neighbour-joining tree based on simple matching distances constructed from genotype of 201 accessions of the *indica* diversity panel, using 825 SNP markers. Subpopulation 1: traditional lowland *indica*; Subpopulation 2: improved lowland *indica*; Subpopulation 3: traditional lowland varieties from Madagascar; Subpopulation 4: *aus accessions*; m: admixed accessions. Subpopulation numbers followed by “P” refer to accessions that were phenotyped in the framework of the present study.

Based on Structure results, the most likely number of subpopulations was four (data not shown). The assignments of the 201 accessions to one of the four subpopulations, based on the proportion of their inferred ancestry from each subpopulation, are represented on the NJ tree (Fig 1). Subpopulation SP1 (76 accessions) regrouped mostly traditional lowland *indica* varieties from all over the world with a sub-organization on the tree based on geographic origins. Subpopulation SP2 (71 accessions) regrouped improved *indica* varieties mainly from IRRI and AfricaRice breeding programs for lowland cultivation, and a few improved *indica* for upland cultivation. Subpopulation SP3 (19 accessions) was composed of traditional lowland varieties originating from Madagascar belonging to the special group that is found at medium elevation [59]. Subpopulation SP4 (6 accessions) regrouped *aus* varieties from the Indian subcontinent. The remaining 29 accessions were admixed. Among the 167 accessions of the panel, that were phenotyped in the present study, 55 belonged to subpopulation SP1, 66 to SP2, 18 to SP3, 4 to SP4 and 24 were admixed (S1 Fig).

### Phenotypic diversity

**Fig 2:**
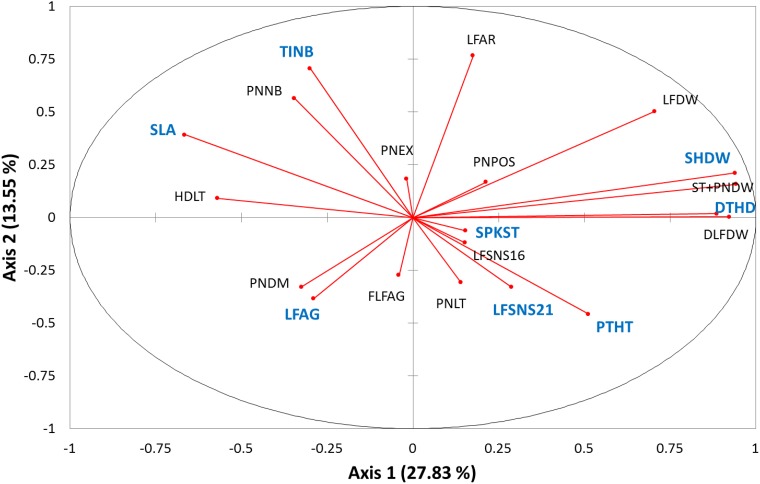
Projection of the contribution of the 20 phenotypic traits on the plan of the first two axes of a Principal Component Analysis. Traits subjected to three methods of GWAS are indicated in blue.

A large variability was observed within the panel for each of the 20 phenotypic traits considered (S2 Fig). The first axis of a PCA performed with these traits including the 167 accessions accounted for 27.8% of the total variation and opposed DTHD, LFSNS and traits related to biomass (LFDW, DLFDW, SHDW, PNDW) to SLA and HDLT (Fig 2). Traits most contributing to the second axis (accounting for 13.6% of total variation) were mainly related to plant architecture: PTHT, TINB, LFAR, FLFAG, LFAG, PNEX and PNDM. SPKST had a rather small contribution to axes 1, 2 and 3 of the PCA, much higher contribution to axes 4, 5, 6 (about 11%) and axis 7 (52%). However, the axis 7 accounted for 4.5% of total variation, only.

The FDA using the membership in subpopulations defined by the structure analysis as categorical variables showed that those subpopulations differed significantly for the combinations of the 20 phenotypic traits considered (S3 Fig). The first axis of the FDA, accounting for 77.7% of total variation, opposed subpopulation SP1, composed of landraces with high plant stature and biomass, long duration, and exhibiting rapid senescence under high temperature treatment, to SP2 composed of improved varieties with shorter stature, smaller biomass and shorter duration. The second axis, accounting for 11.4% of total variation, opposed SP3 and SP4 that had contrasted SPKST, FLFAG, LFAG, LFAR, PNLT and PNNB.

**Fig 3:**
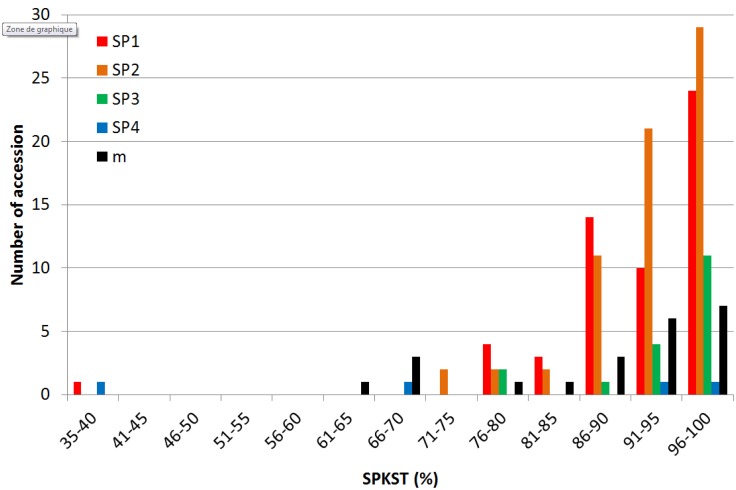
Distribution of spikelet sterility rate among the four Structure subpopulations composing the diversity panel. SP1: traditional lowland *indica*; SP2: improved lowland *indica*; SP3: traditional lowland varieties from Madagascar; SP4: *aus* accessions; m: admixed.

Compared to other traits, SPKST, due to severe heat treatment during the process of anthesis, had a minor discriminating power among the four subpopulations (Fig 3). Indeed, while the mean spikelet sterility under control treatment (SPKSTc) averaged 24% with standard deviation of 13%, the mean SPKST under heat stress averaged 92% with standard deviation of 9%. Individual SPKST under control treatment and heat treatment are going in the same direction, but the overall relationship between SPKSTc and SPKST was very loose, r^2^ = 0.029. The lowest SPKST were observed in N22, an *aus* variety from India, and Peh Kuh, an *indica* landrace from Taiwan. Some improved accessions such as B6144-MR-6-0-0 (from Indonesia), IR 22 and IR2344-P1PB-9-3-2B from the Philippines, and Andy 11 from Mali, exhibited medium tolerance.

### Association analyses

#### Association mapping using single-marker-based regression

Results of association analysis for all phenotypic traits submitted to Sm-GWAS are presented in S4 Table. Results for the eight most discriminant traits among our diversity panel are presented in Table 1. At least one significant association was detected for all of the 20 phenotypic traits except PNLT and PNPOS. The number of SNP significantly associated with those 18 traits varied from one (for PNNB) to 54 (for LFAG). A total of 183 significant associations (p-value < 1e-05) were detected, of which two with p-value <1e-09, three with 1e-09<p-value <1e-08, nine with 1e-08<p-value <1e-07, and 19 with 1e-07<p-value <1e-06. The number of independent loci (i.e. cluster of SNPs with distance between two consecutive significant SNPs below 200kb, corresponding to an average r^2^ >0.2) associated with each trait varied from one (PNEX, TINB, PNNB, DTHD and SHDW) to 15 (for SPKST) and reached a total of 95 (S4 Table). For instance, for PTHT and SPKST, the total number of significant SNPs was five and 27, respectively, and the number of independent loci was two and 15, respectively. These loci were composed of 1–5 SNPs not always adjacent, with p-value ranging between 1e-05 and 1e-09.

**Table 1:** Results of single marker based association analysis (Sm-GWAS) for eight phenotypic traits, and characteristics of the loci significantly associated with each trait.

Chr	Site	PTHT	LFAG	TINB	SLA	DTHD	SHDW	LFSNS 21	SPKST	MAF	Marker R2	Marker effect
1	5 721 734							8.E-06		0.08	0.05	-0.03
1	5 815 041							3.E-05		0.08	0.05	-0.02
1	27 161 527		4.E-05							0.12	0.10	-12.41
1	27 196 502		9.E-06							0.1	0.11	-15.17
1	27 251 511		4.E-08							0.07	0.17	-24.52
1	27 262 971		9.E-05							0.08	0.09	-15.59
1	27 718 131		2.E-05							0.05	0.10	-16.76
1	27 941 462		4.E-06							0.08	0.12	-16.08
1	30 476 158								6.E-05	0.37	0.06	0.05
1	33 007 138								7.E-05	0.44	0.09	-0.08
1	34 906 121								6.E-06	0.04	0.12	0.21
1	35 001 790								6.E-06	0.04	0.12	0.21
1	35 080 336								2.E-07	0.03	0.15	0.26
1	38 434 998	9.E-05								0.49	0.08	-15.74
1	38 999 211	2.E-07								0.35	0.14	24.57
1	39 032 128	2.E-07								0.35	0.14	24.57
1	39 264 931	7.E-05								0.19	0.08	-13.97
1	39 563 606	2.E-05								0.43	0.10	13.90
2	891 822								4.E-06	0.05	0.12	0.15
2	6 086 495							9.E-05		0.35	0.06	0.01
2	8 342 506		8.E-05							0.08	0.09	13.04
2	8 442 473		5.E-05							0.07	0.09	-13.89
2	8 449 912		3.E-06							0.08	0.13	-15.18
2	8 454 752		5.E-05							0.07	0.09	-13.89
2	34 882 131							9.E-05		0.49	0.05	-0.01
2	34 889 065							9.E-05		0.43	0.10	-0.01
3	137 167							2.E-05		0.16	0.03	0.02
3	1 177 466					9.E-05				0.35	0.08	9.28
3	9 308 870				6.E-06					0.37	0.11	-26.75
3	9 324 606				2.E-05					0.34	0.10	24.77
3	9 363 021				6.E-05					0.35	0.09	21.98
3	9 896 090				1.E-05					0.32	0.08	59.14
3	10 147 632		5.E-07							0.05	0.14	-19.74
3	10 197 954		2.E-06							0.08	0.13	-15.37
3	12 537 441								3.E-09	0.04	0.19	0.25
4	2 533 498					1.E-05				0.22	0.10	8.86
4	6 069 831								9.E-05	0.16	0.09	0.09
4	16 718 536		9.E-05							0.03	0.09	-17.91
4	17 876 533								7.E-05	0.11	0.09	0.10
4	25 760 519							9.E-06		0.07	0.04	0.01
4	21 450 464						7.E-05			0.04	0.08	-30.64
4	30 227 164							9.E-05		0.17	0.04	-0.04
4	30 483 968		7.E-05							0.05	0.09	-16.37
4	30 571 650		6.E-05							0.06	0.09	-15.04
4	30 573 319		6.E-07							0.05	0.14	-22.99
4	30 575 948		6.E-07							0.05	0.14	-22.99
4	30 580 954		6.E-07							0.05	0.14	-22.99
4	30 585 343		6.E-07							0.05	0.14	-22.99
4	30 598 452		6.E-07							0.05	0.14	-22.99
4	30 607 537		6.E-07							0.05	0.14	-22.99
4	30 610 597		6.E-07							0.05	0.14	-22.99
4	31 616 428					5.E-05				0.47	0.09	6.44
4	31 751 802					4.E-06				0.49	0.12	7.30
4	31 764 040					2.E-05				0.3	0.10	7.75
5	147 944			1.E-06						0.38	0.12	-4.68
5	165 296			8.E-05						0.42	0.08	-3.67
5	692 983							9.E-05		0.17	0.03	-0.02
5	3 664 582		9.E-10							0.05	0.20	-27.46
5	3 674 037		9.E-10							0.05	0.20	-27.46
5	3 765 056		9.E-10							0.05	0.20	-27.46
5	3 977 568		2.E-05							0.06	0.10	-14.83
5	4 131 283		7.E-06							0.07	0.11	-16.17
5	4 146 770		7.E-06							0.07	0.11	-16.17
5	4 188 405		2.E-06							0.05	0.12	-17.89
5	4 198 477		2.E-06							0.05	0.12	-17.89
5	4 254 037		2.E-06							0.05	0.12	-17.89
5	4 266 382		2.E-06							0.05	0.12	-17.89
5	4 417 332		3.E-08							0.04	0.17	-24.67
5	4 484 555		3.E-06							0.04	0.12	17.48
5	4 554 886		1.E-05							0.05	0.11	-15.74
5	4 620 602		2.E-06							0.05	0.12	-17.89
5	8 970 765		5.E-05							0.07	0.09	13.63
5	22 627 608								7.E-05	0.04	0.09	- 0.19
5	22 836 374								9.E-05	0.02	0.09	- 0.29
5	22 872 767								9.E-05	0.02	0.09	- 0.29
5	23 074 249								9.E-05	0.02	0.09	- 0.29
5	23 213 365								9.E-05	0.02	0.09	- 0.29
6	2 382 723				9.E-05					0.04	0.09	59.14
6	2 859 743				4.E-05					0.13	0.10	39.82
6	3 081 327								4.E-05	0.05	0.10	0.18
6	3 191 839				9.E-05					0.35	0.09	-26.46
6	3 359 591								1.E-05	0.06	0.11	0.14
6	3 437 953								5.E-07	0.05	0.14	0.19
6	28 956 397					4.E-06				0.08	0.12	-11.96
7	3 538 451							9.E-05		0.19	0.02	0.04
7	5 277 899		7.E-07							0.03	0.14	-25.83
7	5 387 715		7.E-07							0.03	0.14	-25.83
7	5 388 753		4.E-05							0.04	0.10	-25.83
7	5 424 303		4.E-05							0.04	0.10	-19.90
7	5 457 657		7.E-07							0.03	0.14	-19.90
7	5 611 869		5.E-05							0.04	0.09	-17.17
7	5 643 230		6.E-05							0.04	0.09	-16.09
7	8 833 570							6.E-05		0.03	0.13	-0.01
7	15 887 872							9.E-05		0.41	0.01	0.01
7	19 592 109				1.E-05					0.04	0.01	-45.64
7	19 948 291				2.E-05					0.02	0.10	64.93
7	22 976 125		1.E-05							0.05	0.11	-18.20
7	23 077 968		3.E-05							0.05	0.10	-16.39
7	23 495 097		1.E-05							0.05	0.11	-18.20
7	23 523 728		7.E-06							0.06	0.11	-19.79
7	29 322 083								9.E-05	0.2	0.08	- 0.08
8	18 993 843		1.E-05							0.11	0.11	-12.96
8	19 029 370		5.E-05							0.07	0.10	-13.85
8	26 037 764						9.E-05			0.05	0.07	-26.02
8	26 047 920						3.E-05			0.04	0.08	-28.93
9	20 936 773							9.E-05		0.4	0.03	0.03
9	20 951 734							8.E-05		0.4	0.04	0.03
10	15 924 661		9.E-05							0.07	0.09	-13.40
10	17 237 925							9.E-05		0.19	0.02	0.03
10	17 381 269							9.E-05		0.17	0.03	0.03
10	20 930 075								6.E-05	0.13	0.09	0.09
10	21 182 537								8.E-06	0.17	0.08	0.07
11	442 456								5.E-05	0.27	0.08	- 0.06
11	19 036 381				2.E-05					0.1	0.10	-35.65
11	19 474 124				3.E-05					0.12	0.10	-32.26
11	20 139 623								8.E-05	0.06	0.09	- 0.14
11	21 860 824				9.E-05					0.1	0.08	45.48
11	21 862 228				4.E-05					0.1	0.09	47.87
12	3 298 469		5.E-06							0.08	0.12	-16.83
12	3 337 493		5.E-06							0.08	0.12	-16.13
12	3 418 976		3.E-05							0.05	0.10	16.13
12	6 136 693							9.E-05		0.25	0.03	0.12
12	24 182 554								2.E-05	0.03	0.10	0.22
12	25 624 918								3.E-08	0.03	0.25	0.26
12	25 666 758								2.E-05	0.05	0.11	0.14
12	25 672 735								3.E-09	0.04	0.19	0.22
12	25 675 740								3.E-09	0.04	0.19	0.22

PTHT: Plant height; LFAG: Angle of the leaf immediately below the flag leaf; TINB: number of tillers; DTHD: time of flowering; SLA: Specific leaf area; SHDW: shoot dry weight; LFSNS 21: Leaf senescence; SPKST: Spikelet sterility.

The contribution of individual significant SNPs to the total variance of the trait considered (marker R2) varied from 3% (one of the SNPs associated to LFDW) to 25% (one of the SNPs associated to SPKST). Among the 183 significant SNPs, 43% had marker R2>10%. Among the 27 SNPs significantly associated with SPKST, 12 had marker R2>10%. The amplitude of allelic effects at individual significant SNPs was also high for almost all traits considered. For instance, it varied from -13 to +24 cm for PTHT and from -29% to 26% for SPKST. The average MAF of the significant SNPs was 0.18 but varied highly according to traits (from 0.04 for FLFAG to 0.41 for TINB). The average MAF was 0.36 for PTHT and only 0.09 for SPKST.

#### Association mapping using haplotype approach

As expected, the haplotype approach detected a much higher number of individual haplotypes associated with each of the eight phenotypic traits considered (Table 2). The total number of significant haplotypes was 883 for the eight traits considered, against 127 SNP loci with Sm-GWAS. The number of significant individual haplotypes was 46 for PTHT and 160 for SPKST against five and 27 detected with Sm-GWAS. The number of independent loci (i.e. cluster of haplotypes with distance between two consecutive haplotypes below 200kb) associated to each trait was also much higher. It varied from 11 for PTHT and SHDW to 52 for SPKST. The mean size of the independent loci varied from 35 to 462 kb (average of 200 kb) for PTHT and from 18 to 925 kb (average of 254 kb) for SPKST. Mean MAF of SNPs composing individual significant haplotypes varied much less among the eight traits (from 0.18 for SLA to 0.27 for SHDW) than the one observed for Sm-GWAS. The mean MAF for SNPs composing each of 11 independent loci associated with PTHT varied from 0.09 to 0.35 (average of 0.19). It varied from 0.04 to 0.31 (average of 0.19) for the 52 independent loci associated with SPKST.

**Table 2:** Co-localization of the loci significantly associated with one of the eight phenotypic traits as detected by three GWAS methods.

		Traits									
GWAS methods	Main features	PTHT	LFAG	TINB	SLA	DTHD	SHDW	LFSNS21	SPKST	Total	Mean
Sm-GWAS	Number of significant SNPs (P-value < 1e-05)	5	54	2	13	6	3	17	27	127	
	Number of Independent loci	2	14	1	5	4	2	13	15	56	
	Mean MAF of significant SNPs	0.36	0.06	0.41	0.18	0.27	0.04	0.26	0.09	-	0.21
Hap-GWAS	Number of significant haplotypes (RLRT > 8)	46	107	70	146	169	31	154	160	883	
	Number of independent loci	11	19	10	33	28	6	34	52	193	
	Mean MAF of SNPs composing each independent locus	0.21	0.19	0.20	0.18	0.19	0.27	0.21	0.19	-	0.21
BL-GWAS	Number of significant SNPs	75	42	34	69	57	42	55	86	460	
	Number of independent loci	29	13	17	31	43	23	39	27	222	
	Number of significant SNPs after robustness test	35	-	-	-	-	-	-	23	-	
	Number of independent loci after robustness test	14	-	-	-	-	-	-	12	-	
	Mean MAF	0.36	0.34	0.38	0.36	0.31	0.35	0.38	0.30	-	0.35
Colocalization of significant SNPs	SNPs detected by Sm-GWAS and Hap-GWAS	5	4	0	1	6	3	3	14	36	
	SNPs detected by Sm-GWAS and BL-GWAS	5	1	0	8	6	3	5	10	38	
	SNPs detected by Hap-GWAS and BL-GWAS	9	0	2	0	7	7	6	6	37	
	SNPs detected by Sm-GWAS, Hap-GWAS and BL-GWAS	5	0	0	0	6	3	3	4	21	
Colocalization of significant independent loci	Loci detected by Sm-GWAS and Hap-GWAS	2	2	0	1	5	2	3	10	25	
	Loci detected by Sm-GWAS and BL-GWAS	2	0	0	3	4	1	1	6	17	
	Loci detected by Hap-GWAS and BL-GWAS	5	1	2	0	6	2	4	5	25	
	Loci detected by Sm-GWAS, Hap-GWAS and BL-GWAS	2	0	0	0	4	1	1	4	12	
Colocalization of significant independent loci with QTLs reported in the literature	Number of QTLs in the literature	68	13	29	7	75	51	83	54	380	
	Sm-GWAS and QTLs from literature	5	0	2	0	6	2	4	9	28	
	Hap-GWAS and QTLs from literature	14	5	3	2	14	8	24	17	87	
	BL-GWAS and QTLs from literature	21	1	1	1	18	5	11	18	76	
	Sm-GWAS, Hap-GWAS & QTLs from literature	6	0	0	0	6	2	3	4	21	
	Sm-GWAS, BL-GWAS & QTLs from literature	5	0	0	0	6	1	0	4	16	
	Hap-GWAS, Hap-GWAS & QTLs from literature	7	0	0	0	6	1	3	1	18	
	Three GWAS methods and QTLs from literature	5	0	0	0	6	1	0	1	13	

PTHT: Plant height; LFAG: Angle of the leaf immediately below the flag leaf; TINB: number of tillers; DTHD: time of flowering; SLA: Specific leaf area; SHDW: shoot dry weight; LFSNS 21: Leaf senescence; SPKST: Spikelet sterility.

#### Association mapping using Bayesian lasso model

In spite of a very stringent significance threshold based on the results of permutation tests, simultaneous fitting of all markers using Bayesian lasso model detected a large number of SNPs with effects significantly different from zero. These 460 SNPs represented a total of 222 independent loci for the eight phenotypic traits considered (Table 2). The model detected 75 SNPs with significant effect corresponding to 29 independent loci for PLHT (S5 Table) and 86 SNPs with significant effect corresponding to 27 independent loci for SPKST (S6 Table). The robustness test reduced the number of SNPs with high effect to 35, which corresponded to 14 independent loci, for PTHT (S5 Table) and to 23, which corresponded to 12 independent loci, for SPKST (S6 Table). Mean MAF among the 460 significant loci was much higher than in the case of the Sm-GWAS and varied from 0.30 for SPKST to 0.38 for TINB.

#### Congruence of results between the three association analysis methods

The number of significant independent loci common to the three GWAS methods was trait-dependent. For instance, while in the case of PTHT, the two loci detected by Sm-GWAS were also detected by Hap-GWAS and by BL-GWAS, in the case of SPKST, among the 15 significant loci detected under Sm-GWAS, only 10 were detected under Hap-GWAS and 6 under BL-GWAS. And among these loci, only four were common to the three GWAS methods. Interestingly, as shown in the case of PTHT, each of the five individual SNPs detected under Sm-GWAS belongs to a cluster of 5–10 SNPs forming haplotypes detected as significantly associated with PTHT by the Hap-GWAS method.

The proportions of independent loci under Sm-GWA also detected by Hap-GWAS or BL-GWAS were, respectively, 7% and 2% for LFAG, 0% and 0% for TINB, 0% and 62% for SLA, 100% and 100% for DTHD, and for SHDW, 12% and 29% for LFSNS21, and 52% and 37% for SPKST. Overall, among the 56 independent loci under Sm-GWAS, 25 were common with the 193 significant independent loci mapped with Hap-GWAS, and 14 were common with the 222 significant independent loci mapped with BL-GWAS. The number of significant loci common to Hap-GWAS and BL-GWAS was 25. Finally, only 12 significant independent loci were common to the three GWAS methods, of which two for PTHT and four for SPKST.

### Characteristics of loci associated to target traits

#### Co-localization with QTLs reported in the literature

We inventoried a total of 380 QTLs detected in progenies of biparental crosses for our eight target traits (S7 Table). The proportion of these QTLs co-localizing with at least one significant independent locus was 74% for Sm-GWAS, 23% for Hap-GWAS and 20% for BL-GWAS. The co-localization dropped below 5% for loci common to two or three methods.

**Fig 4:**
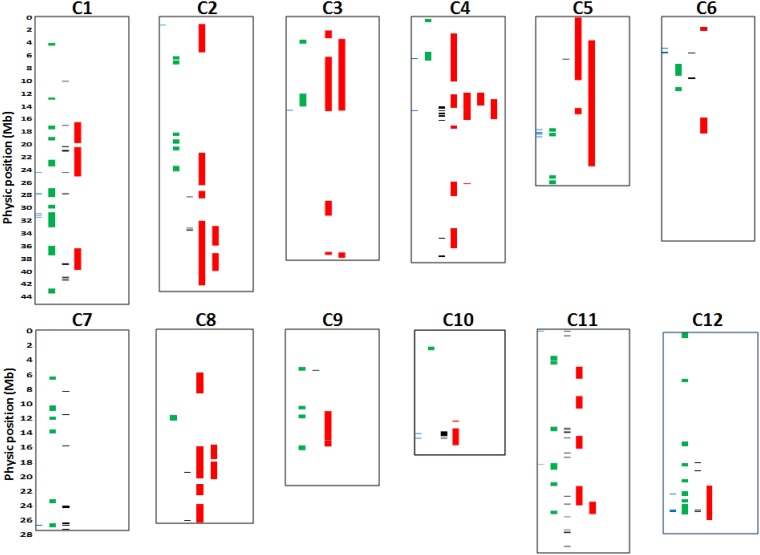
Co-localization of significant SNPs and haplotypes detected by the three GWAS methods with QTL for heat tolerance during the reproductive phase detected in biparental crosses. Blue: Sm-GWAS, Green: Hap-GWAS, Black: BL-GWAS and Red: QTL.

The chromosomic localization of the 54 QTLs inventoried for SPKST under high temperature and the localization of significant independent loci detected with each of the three GWAS methods is summarized in Fig 4. Among the 15 significant loci associated with SPKST under Sm-GWAS, nine (60%) colocalised with at least one QTL detected in progenies of biparental crosses. The proportion was 33% for the 52 independent loci under Hap-GWAS, 66% for the 27 independent loci under BL-GWAS, and 42% for the 12 independent loci under BL-GWAS after robustness test. Specially, significant independent loci detected by at least two GWAS methods colocalised with the most consistent QTL mapped on chromosome 4 for SPKST under high temperature in the progenies of both IR64/N22 and IR64/Giza178 crosses. Likewise, significant loci colocalised also with several other QTLs mapped in the progenies of these crosses (Fig 4; S6 Table).

#### Genomic localization

To examine the genomic localization of the independent loci, we identified the positions of the most significant SNPs by MSU database search and gene annotation. Among a total of 471 such SNPs detected by the three GWAS methods for the eight target traits, 51% were located in intergenic regions, 24% in introns, 11% in exons with synonymous coding effects, 7% in exons with non-synonymous coding effects, 6% in UTR-3 regions and 0.2% in stop-gained sites (data not shown). These proportions were similar to those observed among all the 13,160 SNPs used for GWAS. Likewise, little variations were observed for these proportions among traits and among GWAS methods (data not shown). Thus, we limited further analysis of localization to the cases of PTHT and SPKST and to loci common to at least two GWAS methods. As our diversity panel included semi-dwarf accessions, we used the PTHT case to check the power of our data and GWAS methods to detect the semi-dwarfing loci *Sd1*, OsGA20ox2, (Loc_Os01g08838, Chr.1: 38,382,385–38,385,469).

#### Genomic localization of loci associated with PTHT

The two independent loci associated with PTHT under two or three GWAS methods were both located downstream from the semi-dwarfing gene *Sd1*. The closest locus, represented by SNP S01_38434998, was located 52.6 kb downstream of *Sd1*. The marker affected PTHP by 15.8 cm, and had a R2 of 0.08. The second locus, SNP S01_38999211, was located 617 kb downstream of *Sd1*. The marker affected PTHP by 25.3 cm, and had a R2 of 0.13. Despite a distance of 564 kb, linkage disequilibrium between the two SNP loci was high (r^2^ = 0.5), and alleles at the two loci formed three haplotypes corresponding to significantly different PTHT (p<0.0001). The three haplotypes also significantly distinguished the four sub-populations identified within the panel: while the haplotype AG (mean PTHT = 145 cm) and AT (mean PTHT = 99 cm) were present exclusively in sub-populations SP1 (traditional lowland varieties) and SP3 (improved lowland varieties), respectively, the haplotype GG (mean PTHT = 141 cm) was shared by subpopulations SP1, SP2 and SP4. Thus, (i) the power of our data and GWAS methods in detecting loci with rather high effects was confirmed despite the superposition of the panel structure and the distribution of the target traits within the panel, and (ii) the rule we used to define “independent loci” (distance of 200 kb between adjacent SNP, corresponding in average to a r^2^ > 0.2) did not apply in the vicinity of *Sd1* locus subject to high selection pressure within the rice breeding programs.

#### Genomic localization of loci associated with SPKST

Among the 22 SNPs significantly associated with SPKST under two or three GWAS methods, 13 were located in annotated genes and 7 in intergenic regions. Among the annotated genes, at least 7 were described as involved in plant response to abiotic stresses or to gametophyte development (Table 3). Survey of genome annotation within a surrounding interval of 100 kb (50 kb downstream and 50 kb upstream), each of the 22 significant SNPs led to the identification of at least one gene with product involved in (i) plant response to abiotic stresses and transcription regulation such as heat shock proteins (SHP), wall associated kinase (WAK), wall receptor-like protein kinase (WRKY), F-box domain containing protein, serine carboxypeptidase homologue, (ii) male–female interactions in plant sexual reproduction, gametophyte development, and senescence such as DEFL peptides, F-box, and the seed maturation protein PM23, (iii) cell division such as the SNF2 family N-terminal domain containing protein, the TBC domain containing protein, regulator of chromosome condensation, (iv) osmotic adjustment such as the cation/H^+^ exchanger OsCHX15 (Table 3). For instance, in the case of the significant SNPs colocalizing with the major QTLs mapped in IR64/N22 and IR64/Giza178 crosses, genome survey led to a F-box/LRR-repeat protein and OsMADS21 (involved in flower development) for qHTSF1.1, to OsWAK43 and OsWAK44 (involved in gametophyte development) for qHTSF4.1, and to OsFBX187 and OsIAA20 for qHTSF6.1.

**Table 3:** Results of the survey of genome annotation within an interval of 100 kb (50 kb downstream and 50 kb upstream) around each locus associated with SPKST under 2 or 3 GWAS methods.

chr	Position (bp)	GWAS methods			QTL	Candidate genes and associated functions
		Sm	Hap	BL		
1	30 476 158	1		1	q01.2	Os01g53020 (heat shock protein DnaJ)
1	33 007 138	1	1	1		Os01g57110 (SNF2 family N-terminal domain containing protein)
1	34 906 121	1	1			Os01g60340 (NTMC2Type1.1 protein); Os01g60490 (WRKY22)
1	35 001 790	1	1			Os01g60520 (WRKY116)
1	35 080 336	1	1			Os01g60640 (WRKY21)
4	6 069 831	1	1		q04.1	Os04g11165 (gamma-thionin family domain containing protein); Os04g11130 (DEF9—Defensin and Defensin-like DEFL family)
**4**	**17 876 533**	**1**		**1**	**q04.2**	**Os04g29960 (OsWAK43—OsWAK receptor-like protein kinase); Os04g29990 (OsWAK44—OsWAK receptor-like protein kinase)**
5	22 627 608	1	1		q05.2	Os05g38570 (riboflavin biosynthesis protein ribAB, chloroplast precursor); Os05g38550 (ubiquitin-conjugating enzyme)
5	22 872 767	1	1		q05.2	Os05g39600 (ATCHX15, putative); Os05g39540 (metal cation transporter, putative, expressed)
5	23 074 249	1	1		q05.2	Os05g39330 (retrotransposon protein, putative)
6	3 437 953	1		1		Os06g07180 (expressed protein); Os06g07190 (RNA polymerase Rpb7, N-terminal domain containing protein)
7	29 312 153		1	1		
7	29 322 083	1	1	1		Os07g49000 (DNAJ heat shock N-terminal domain-containing protein, putative); Os07g49010 (OPBP1B—Similar to DNA replication protein)
9	8 156 835		1	1		Os09g13870 (lipid phosphatase protein)
10	20 930 075	1		1	q10.2	Os10g39200 (expressed protein); Os10g39190 (B3 DNA binding domain containing protein)
10	21 182 537	1		1	q10.2	Os10g39640 (expansin precursor, putative); Os10g39620 (ubiquitin family protein)
11	442 456	1		1		Os11g01872 (HEAT repeat family protein, putative); Os11g01820 (ATCHX, putative, expressed)
11	15 692 357		1	1		Os11g27264 (OsSCP60—Putative Serine Carboxypeptidase homologue)
11	15 712 256		1	1		Os11g27329 (OsSCP62—Putative Serine Carboxypeptidase homologue)
11	20 139 623	1	1			Os11g34360 (lung seven transmembrane domain containing protein); Os11g34320 (Regulator of chromosome condensation)
12	24 182 554	1	1		q12.1	Os12g39320 (DUF221 domain containing protein); Os12g39200 (Seed maturation protein PM23)
12	25 624 918	1	1	1	q12.1	Os12g41350 (meiotic asynaptic mutant 1); Os12g41300 (OsFBX462—F-box domain containing protein)
12	25 666 758	1	1		q12.1	Os12g41450 (F-box domain containing protein)
12	25 672 735	1	1	1	q12.1	Os12g41400 (Eukaryotic translation initiation factor 2 subunit gamma)
12	25 675 740	1	1		q12.1	Os12g41410 (Receptor-like protein kinase homolog RK20-1)

#### Haplotypes associated with SPKST in our panel and within the *O*. *sativa* major groups

Haplotype construction with the genotype of our 167 accessions at the 22 SNPs’ loci associated with SPKST under at least 2 GWAS methods led to two major haplotypes with significantly different spikelet sterility (80% and 94%, p <0.001). The two most tolerant accessions of our panel (N22 and Peh Kuh) belonged to the same haplotypic cluster and, despite their membership to two different genetic groups, shared the same allele at 21 SNPs out of 22 (S4 Fig).

To explore the genetic diversity at the vicinity of loci associated with SPKST within the *O*. *sativa* major groups, we extracted genotypic data from the IRIC database (http://oryzasnp.org/iric-portal) that hosts the data of 3 000 rice genomes: 3KRG project. Haplotype construction within the accessions of the 3KRG simultaneously using genotypes at the 22 significant SNPs or the 15 corresponding independent loci, did not yield simple and readable patterns. In contrast, haplotypes at the vicinity of individual significant loci were much more informative. For instance, the 19 SNPs extracted from the interval of 100 bp surrounding the significant SNP D04_17876533, that colocalised with qHTSF4.1 on chromosome 4, formed 5 major haplotypes. And while four of these haplotypes were related chiefly to membership to genetic group (*indica*, *japonica*, *aus*, or *aromatic*), the fifth one assembled accessions from *indica* and *japonica* groups, and almost all *aus* accessions. This haplotype included heat tolerant accessions common to our diversity panel such as N 22, Taichung native-1, IR22 and B6144 (S5 Fig). Likewise, the 510 SNPs available in Loc_OS04g29960 (OsWAK43), with which D04_17876533 colocalised, formed 5 major haplotypes. One of these haplotypes clustered a large number of accessions from different genetic groups and hosted the heat tolerant accessions common to our diversity panel (S5 Fig). Haplotype analysis at the vicinity of the other SPKST-associated loci led to similar patterns.

## Discussion

The aim of this work was to explore the phenotypic diversity for rice spikelet sensitivity to high temperature, and the associated allelic variations, and to draw a unified picture of the genetic bases of this important trait in the context of climate change, by connecting QTLs previously mapped in bi-parental crosses to the underlying genes. Given the recent development of new GWAS methodologies, we also explored the added value of two methods that resort to more complex models than the classical single marker regression to detect SNP loci associated with phenotypic variation. During our phenotyping experiment for spikelet sensitivity to high temperature, several other traits have also been measured. Some of these traits were related to the plant ability to reduce panicle temperature through transpiration, via either large leaf area (LFAR, SLA, GLFDW, DLFDW, LFSNS 16, LFSNS 21, SLA) or appropriate architecture (PTHT, PNPOS, FLFAG, LFAG), others related to plant potential performance (PNNB, TINB, PNLT, PNEX, SHDW, ST+PNDW), and one related to the duration during which the plant may be susceptible to heat (HDLT). Although we could not find any direct relationship between these traits and SPKST, we included them in our GWAS work with the aim of (i) further ascertaining the suitability of our diversity panel for GWAS analysis and (ii) enriching existing QTL data bases for those traits.

The temperature of 38°C applied during 6 consecutive days at the time of flowering was rather severe, leading to high SPKST for a large number of accessions. However, this did not change the ranking of the most tolerant and most susceptible accessions as reported in literature [2].

Our genotypic data of 13,160 SNP provided a reasonably good coverage of genome (average density of one marker per 29 kb) given the average extent of LD of 125–150 kb. Indeed, we may have failed to detect loci associated with our target traits in only nine chromosomic segments free of markers over more than 500 kb.

The 167 accessions of our diversity panel were structured into four subpopulations, the main structuring element being (i) landrace status (subpopulation SP1) versus modern variety (SP2) and the associated tall or semi-dwarf plant stature, (ii) adaptation to specific ecology and cropping system, such as high elevation areas (SP3) and rainfed lowland cropping in low elevation areas (SP4). Given the well-known risk of false-positive associations in such structured panels [60], we systematically included in the association mapping models a fixed effect with design matrix *Q* to account for population structure. Under the three GWAS methods we utilized, the detection of the loci associated with plant height variations resulting from the presence or absence of the semi-dwarfing gene *Sd1* on the long arm of chromosome 1, highlighted the effectiveness of the *Q* matrix in balancing (i) the risks of false positive tests inherent to population structure and (ii) the risk of false negative tests due to the superposition of the distribution of the phenotypic variability for plant height with the panel structure.

An important issue for GWAS is the minimal size of the panel that provides enough power to detect associations of a given size. The power of the association analysis necessary to detect a QTL by testing the marker effect depends on the LD between markers, the proportion of total phenotypic variance explained by the QTL, the size of the population, the allele frequency of the rare allele at this marker and the significance level α set by the experimenter [47]. In the case of Sm-GWAS, the issue of the significance threshold is reflected into the dilemma of balance between (i) the very stringent correction for the number of markers tested using Bonferroni correction to obtain experiment wise p-value and (ii) the fact that tests on the same chromosome are not independent, as close markers are often in linkage disequilibrium with each other as well as with the QTL. We opted for the common practice adopted in the literature of using a p-value of 1e-05, and then tried to confirm the detected association using Hap-GWAS and BL-GWAS methods. However, the implementation of these methods also faces the question of significance threshold for haplotype or individual marker effect to be declared significant. We resorted to permutation test in the case of BL-GWAS, but despite a reasonably high number (1000) of permutations the stringency was low, leading to a large number of significant loci. By contrast, the robustness test proved to be more efficient though the experimenter still had to determine a threshold.

For each of the eight phenotypic traits considered, Hap-GWAS and BL-GWAS detected a much higher number of significant SNPs (seven times and four times more, respectively) and independent loci (3.5 and four times, respectively) than Sm-GWS. In the case of Hap-GWAS, these higher numbers were expected as marker haplotypes can be in higher LD with a QTL than individual markers. And this led to declare as significant SNPs with very low individual contribution to the trait total variance. For instance, the mean marker R2 of the 160 SNPs belonging to haplotypes significantly associated with SPKST was 2.9% against 11.4% for the 27 SNPs detected by Sm-GWAS. A less expected phenomenon was the fact that all significant SNPs detected by Sm-GWAS were not detected by hap-GWAS. For instance, in the case of SNPs associated with SPKST, only 14 out of the 27 were detected by hap-GWAS. This is probably due to the fact that most of the 27 significant SNPs under Sm-GWAS had rather small MAF and the frequency of haplotypes constructed with these SNPs was still smaller. Moreover, in some cases, the distances between the 6 consecutive SNPs serving to haplotype construction were high, leading to low LD between markers composing the haplotype. For instance, in the case of SPKST, while the average MAF among the 14 SNPs detected by both Sm-GWAS and Hap-GWAS was 17%, it was only 11% in the case of the remaining 13 SNPs. Likewise, while the length of the chromosomic segment covered by the haplotype window of 6 SNPs surrounding each of the 14 significant SNPs under the 2 GWAS methods was 100 kb in average, it was 216 kb for the 13 SNPs not detected by Hap-GWAS.

In the case of BL-GWAS, the prior assumptions on the distribution of possible SNP effects (double exponential or Laplace prior) we used proved, afterwards, not to be the most adapted to our data and thus not stringent enough. Robustness test applied to PTHT and SPKST allowed refining the threshold of significance for the SNPs with the greatest contribution to the observed phenotypic variation. The number of SNPs with p <0.05 was 35 (12 independent loci) and 29 (16 independent loci) for PTHT and SPKST, respectively. As expected, under BL-GWAS, SNPs with smaller R2 were declared significant. For both PTHT and SPKST, the mean marker effect was approximately 5% under BL-GWAS against 11% under Sm-GWAS. The significant SNPs included all those detected by Sm-GWAS, for PTHT, and only 10 of the 27 SNPs for SPKST. The major difference between the 10 common and the 17 non-common SNPs was the MAF, with a marker effect of 18% in average for the former and 5% for the latter. Similarly, while the average MAF for the 27 SNPs significantly associated with SPKST under Sm-GWAS was only 10%, it was 29% for the 35 SNPs significant under BL-GWAS. Thus the two methods differed strongly for their sensitivity to MAF.

The conclusions one can draw from the comparison of results obtained from the three GWAS methods are their differential sensitivity to (i) the genetic architecture of the phenotypic trait and its distribution within the diversity panel, (ii) the characteristics of the genotypic data in terms of regularity of markers distribution along the genome and of equilibrium between alleles at individual SNP loci. Thus, confronting the results of several GWAS methods on a given dataset helps guarding against the effects of their sensitivity to specific features of the data. However, this solution is only a first step. The most reliable solution against those risks remains validation in an independent GWAS experiment and population.

We have detected 14 independent loci significantly associated with SPKST under at least 2 GWAS methods. Favorable alleles for some of these loci were present in some accessions of three subpopulations (SP1, SP2, and SP4) of our diversity panel, but only two accessions, one from SP1 (*indica* landrace) and one from SP4 (*aus*) assembled all favorable alleles. Haplotypes analysis at the vicinity of individual significant loci within the accessions of the 3KRG confirmed the fact that favorable alleles for heat tolerance during anthesis are not confined to a few accessions but are rather widespread across different genetic groups of *O*. *sativa*. However the number of accessions bearing the favorable allele for spikelet fertility despite high temperature at all loci is probably limited. Indeed, within the 3KRG panel, construction of haplotypes using simultaneously the genotypes at all significant loci did not yield a simple and readable pattern as it was the case for individual loci and for our own diversity panel.

Among the 14 independent loci associated with SPKST, eight colocalised with QTLs reported in the literature for tolerance to high temperature during reproductive stage. Our GWAS experiment allowed (i) to narrow-down the position of these eight QTLs detected in bi-parental crosses and (ii) to detect six new QTLs. Among the QTLs reported in the literature, qHTSF4.1 was the most consistent as it was detected across different genetic backgrounds, including the progenies of crosses between the two tolerant varieties N22 and Giza178 with the moderately tolerant variety IR64 [30]. The interval for this QTL reported by these authors was 63.5–80.5 cM, approximately corresponding to the physical interval of 15–19 Mb [61]. Our results narrowed down this interval to LOC_Os04g29960, with a significant non-synonymous SNP (D04_17876533). This locus codes for a wall-associated kinase, OsWAK43, and stands at 11.6 kb of a second WAK locus, OsWAK44. The WAKs are a sub-family of receptor-like kinases associated with the cell wall. They have been suggested as sensors of the extracellular environment and triggers of intracellular signals [62]. In *A*. *thaliana*, disruption of WAK function compromises leaf cell expansion and root cell elongation [63]. In rice, several WAKs participate positively and negatively in basal defense against rice blast fungus [64]. Their involvement in response to abiotic stress is also proposed, with the production of reactive oxygen species and of downward redox signaling, which have a vital function in plant growth, development, and stress response [65]. Some WAKs are also involved in rice gametophyte development [66].

Gene families underlying the 14 independent loci associated with SPKST corresponded to functions ranging from sensing abiotic stresses and regulating plant response to those stresses, to cell division and gametophyte development. This is consistent with the differential morpho-physiological and biochemical responses of rice varieties tolerant or susceptible to heat stress during anthesis. The differential responses apply notably to (i) the degree of anther dehiscence and the length of pollen tube, (ii) the pollen moisture content and the rate of pollen survival, and (iii) the pollen membrane stability indices, the pollen total protein concentration, the ratio of protein with high and low molecular mass, and the concentration of heat shock proteins [23, 25]. The first set of differential responses results from the expression of genes involved in cell expansion such as WAK [65]. The second set results from upregulation of genes involved in osmotic adjustment such as CHX as mature pollen desiccates and then rehydrates at germination [67, 68]. The third set results from upregulation of molecular chaperones such as HSP, and HEAT repeat domains, that recognize and bind substrate proteins that are in an unstable state [69, 70], J-proteins that can partner with HSP [71] and F-box proteins that are critical for the controlled degradation of cellular proteins [72]. Of course, all these differential responses are also related to the expression of genes involved in sensing and signaling high temperature such as the WAK family, and of transcription factors regulating cell and plant response to high temperature such as the SNF2 [73] and the WRKY [74] families.

Given the diversity of the biological processes and associated genes involved in rice response to high temperature during the anthesis process, transfer of tolerance from donors such as N22 and Giza178 to elite materials has little chance to be achieved through conventional and/or marker-assisted backcross breeding. Other approaches such as marker-assisted recurrent selection in bi-parental crosses [75] or, even better, genomic selection in dedicated synthetic population [76] need to be considered.

## Supporting information

S1 Fig Unweighted neighbour-joining tree based on simple matching distances constructed from genotype of 201 accessions of the *indica* diversity panel with 825 SNP markers. Subpopulation 1: traditional lowland *indica*; Subpopulation 2: improved lowland *indica*; Subpopulation 3: traditional lowland varieties from Madagascar; Subpopulation 4: *aus accessions*; m: admixed accessions. Accession name followed by “(p)” were phenotyped in the framework of the present study.
(JPG)

S2 Fig Distribution of selected traits within the diversity panel. plant height (PTHT), time of flowering (DTHD), number of tillers (TINB), shoot dry weight (SHDW), specific leaf area (SLA), angle of the leaf immediately below the flag leaf (LFAG), leaf senescence 21 days after flowering (LFSNS 21) and spikelet sterility (SPKST).
(JPG)

S3 Fig Projection of the 167 accessions on the first plan of a factorial discriminant analysis of 20 phenotypic traits, using the membership of the accessions to the 4 subpopulations defined by structures as categorical variables. SP1: traditional lowland *indica*; SP2: improved lowland *indica*; SP3: traditional lowland varieties from Madagascar; SP4: *aus* accessions m: admixed.
(JPG)

S4 Fig Haplotype construction with the genotype of our 176 accessions at the 22 SNP loci associated with SPKST under at least 2 GWAS methods, led to two major haplotypes with significantly different spikelet sterility. Red (SP1): traditional lowland *indica*; Orange (SP2): improved lowland *indica*; Green (SP3): traditional lowland varieties from Madagascar; Blue (SP4): *aus* accessions m: admixed.
(JPG)

S5 Fig Haplotype diversity at the vicinity of the SNP loci associated with SPKST on chromosome 4 (loci *D*04_17876533, colocalizing with *OsWAK44* gene and QTL *qHTSF4*.*1*, within *O*. *sativa*). Haplotype constructed with **(A)** 19 SNP in the interval of 100 pb surrounding *D*04_17876533, for 2060 accessions, and **(B)**: 510 SNP within *Loc_OS04g29960*, for 2773 accessions. Colour code: Red = *indica*; Green = *japonica*; Blue = *aus*; Purple = *aromatic*; *B*lack = admixed.
(JPG)

S1 Table Main characteristics of the 201 accessions of our diversity panel. (XLSX)

S2 Table Variability of marker density and frequency of minor alleles (MAF) along the 12 chromosomes. (XLSX)

S3 Table Variability of decrease of pairwise linkage disequilibrium with distance between markers among the 12 chromosomes. (XLSX)

S4 Table Results of single marker-based association analysis (Sm-GWAS) for 20 phenotypic traits, and colocalization of significant loci with candidate gens. (XLSX)

S5 Table Colocalization of significant loci detected by the three GWAS methods for plant height (PLHT) with QTLs reported in the literature, and other information related to GWAS for PLHT. (XLSX)

S6 Table Colocalization of significant loci detected by the three GWAS methods for Spikelet sterility (SPKST) with QTL reported in the literature, and other information related to GWAS for SPKST. (XLSX)

S7 Table Co-localization of significant SNP detected by the three association analysis methods with QTLs reported in the literature. (XLSX)
