# Supplementary figures and images for: Genome-wide association analysis for heat tolerance at flowering detected a large set of genes involved in adaptation to thermal and other stresses
Source: PLoS One. 2017 Feb 2;12(2):e0171254. doi: 10.1371/journal.pone.0171254 (PMC5289576; doi:10.1371/journal.pone.0171254)

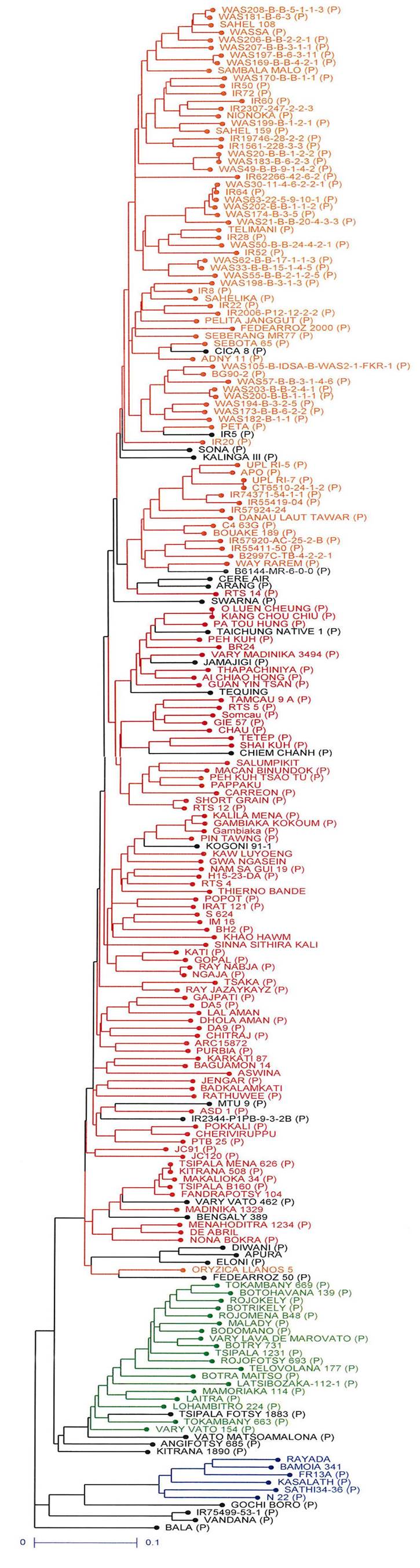

Supplement: S1 Fig — Subpopulation 1: traditional lowland indica; Subpopulation 2: improved lowland indica; Subpopulation 3: traditional lowland varieties from Madagascar; Subpopulation 4: aus accessions; m: admixed accessions. Accession name followed by “(p)” were phenotyped in the framework of the present study. (JPG) [file pone.0171254.s001.jpg]

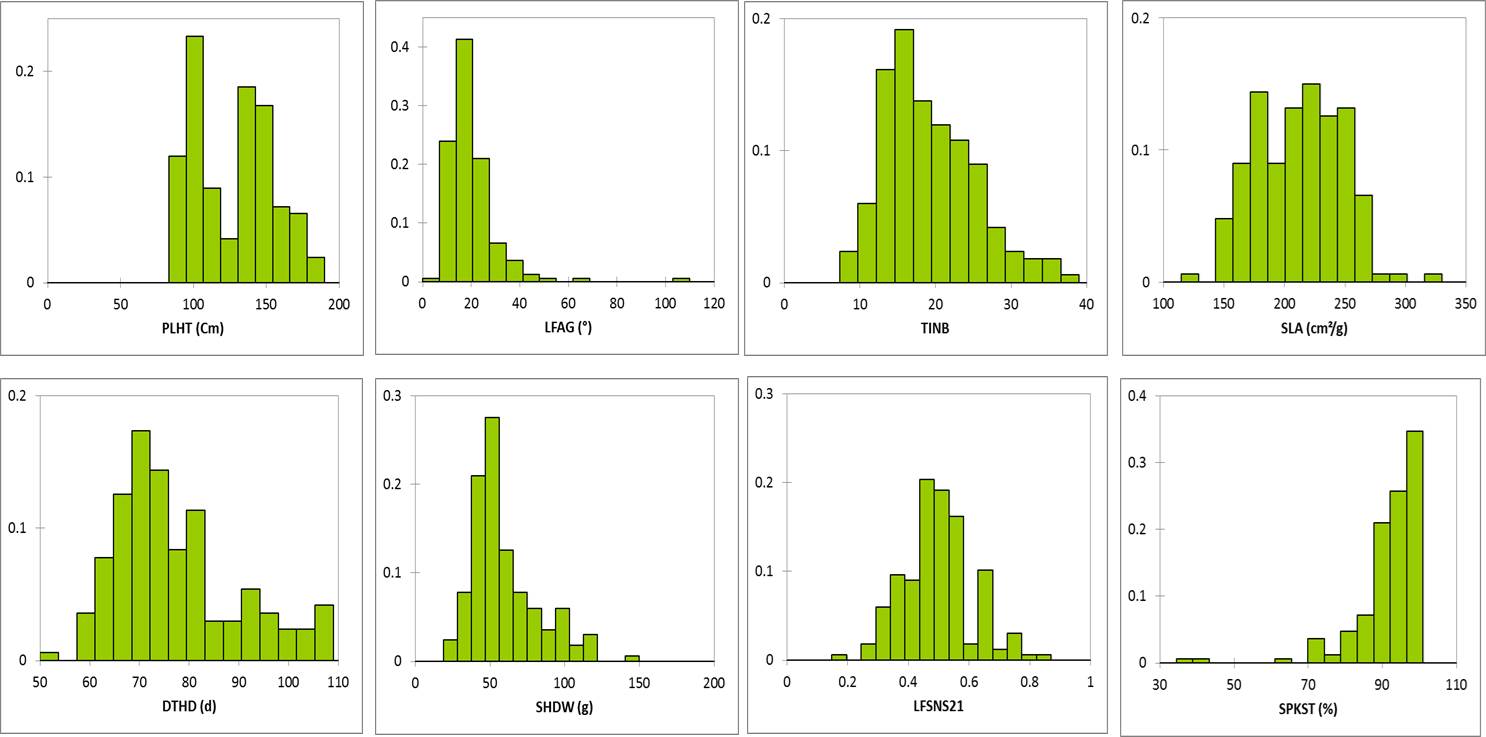

Supplement: S2 Fig — plant height (PTHT), time of flowering (DTHD), number of tillers (TINB), shoot dry weight (SHDW), specific leaf area (SLA), angle of the leaf immediately below the flag leaf (LFAG), leaf senescence 21 days after flowering (LFSNS 21) and spikelet sterility (SPKST). (JPG) [file pone.0171254.s002.jpg]

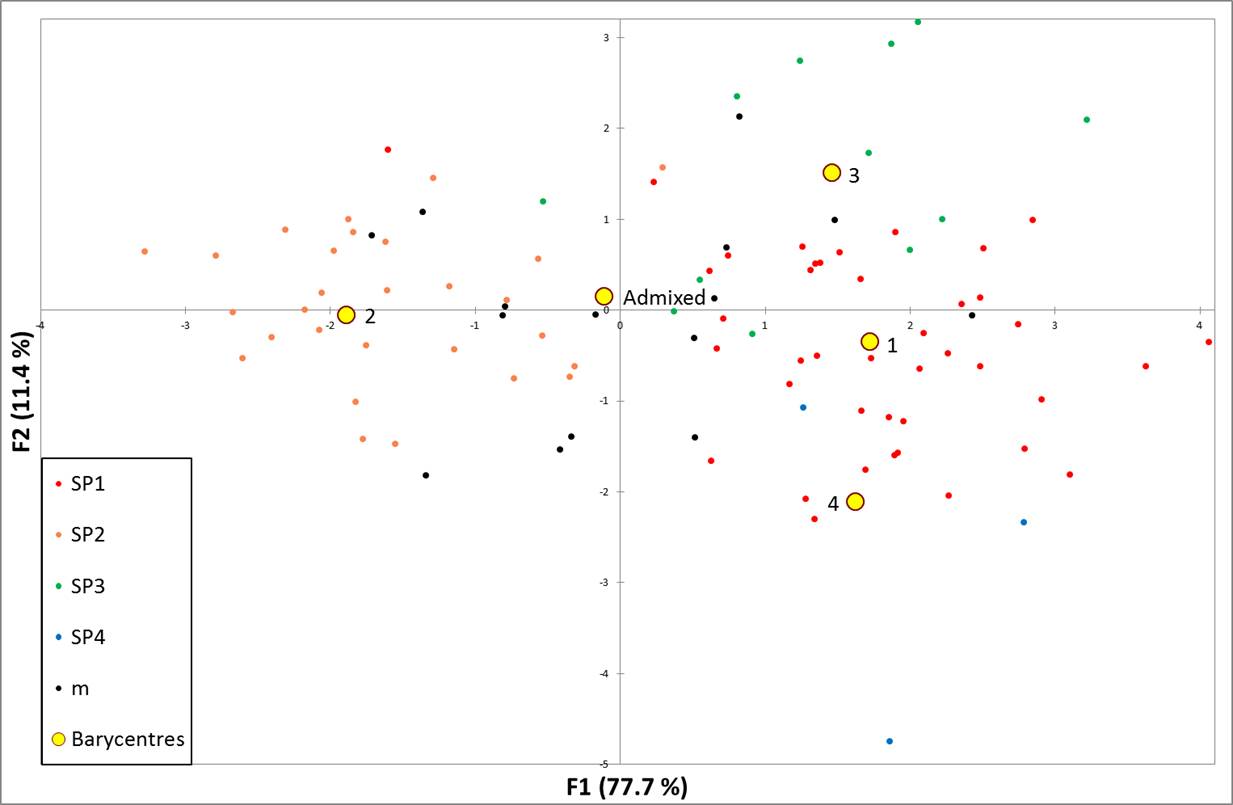

Supplement: S3 Fig — SP1: traditional lowland indica; SP2: improved lowland indica; SP3: traditional lowland varieties from Madagascar; SP4: aus accessions m: admixed. (JPG) [file pone.0171254.s003.jpg]

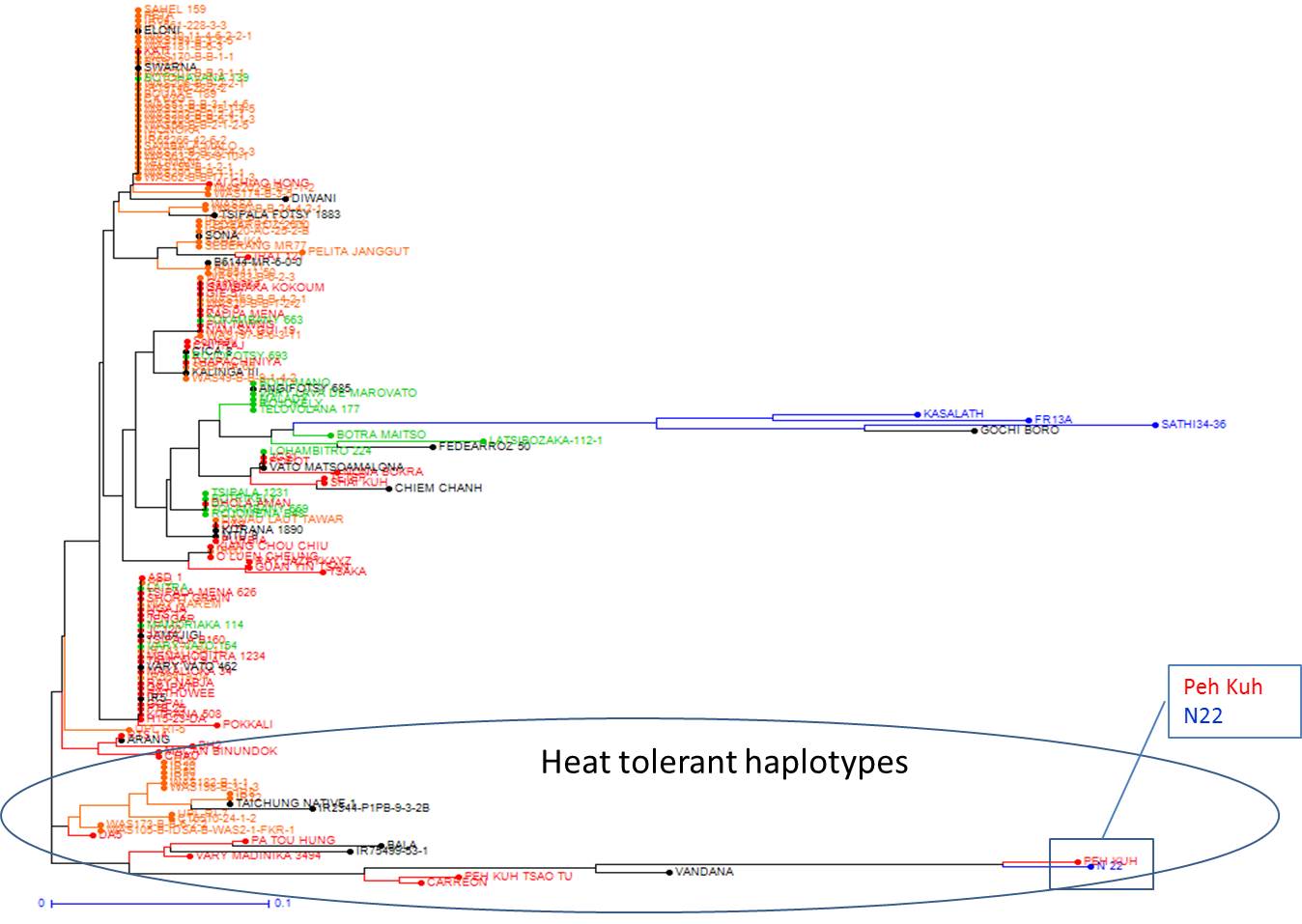

Supplement: S4 Fig — Red (SP1): traditional lowland indica; Orange (SP2): improved lowland indica; Green (SP3): traditional lowland varieties from Madagascar; Blue (SP4): aus accessions m: admixed. (JPG) [file pone.0171254.s004.jpg]

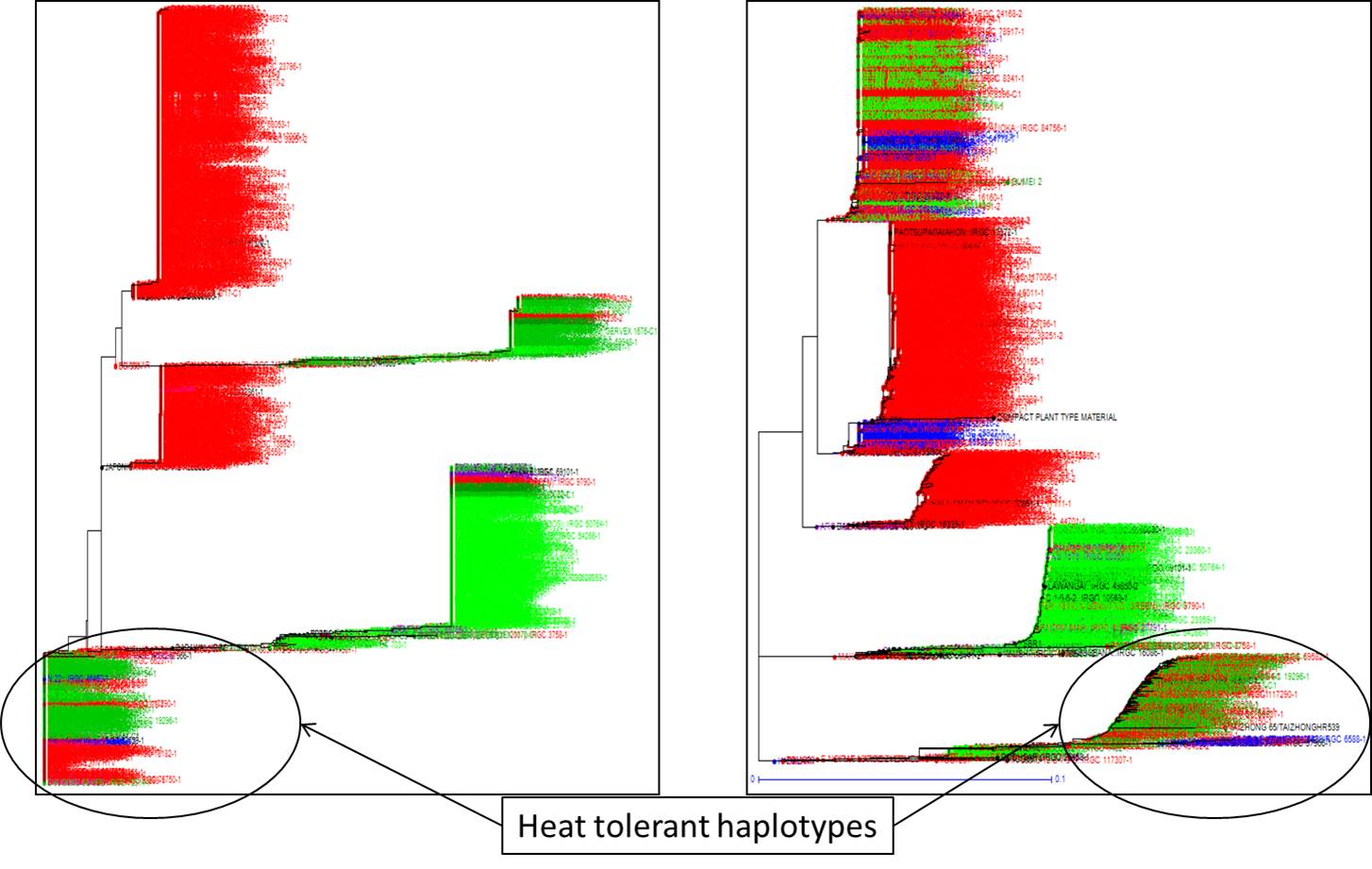

Supplement: S5 Fig — Haplotype constructed with (A) 19 SNP in the interval of 100 pb surrounding D04_17876533, for 2060 accessions, and (B): 510 SNP within Loc_OS04g29960, for 2773 accessions. Colour code: Red = indica; Green = japonica; Blue = aus; Purple = aromatic; Black = admixed. (JPG) [file pone.0171254.s005.jpg]
